# A synthesis of carcass decomposition studies conducted at a tropical (Aw) taphonomy facility: 2013–2022

**DOI:** 10.1016/j.fsisyn.2023.100345

**Published:** 2023-08-08

**Authors:** David O. Carter, Adam Orimoto, Carlos A. Gutierrez, Agathe Ribéreau-Gayon, Emily L. Pecsi, Katelynn A. Perrault, Alexis J.L. Peterson

**Affiliations:** a Laboratory of Forensic Taphonomy, Forensic Sciences Unit, School of Natural Sciences & Mathematics, Chaminade University of Honolulu, Hawaii, USA; b Scientific Investigation Section, Honolulu Police Department, Honolulu, HI, USA; c True Forensic Science, Santiago, Chile; d Department of Chemistry, Biochemistry, and Physics, Université du Québec à Trois-Rivières, Québec, Canada; e Research Group in Forensic Science, Université du Québec à Trois-Rivières, Québec, Canada; f Department of Anatomy, Université du Québec à Trois-Rivières, Québec, Canada; g Department of Environmental Sciences, Université du Québec à Trois-Rivières, Québec, Canada; h Laboratory of Forensic and Bioanalytical Chemistry, Forensic Sciences Unit, School of Natural Sciences & Mathematics, Chaminade University of Honolulu, Hawaii, USA; i Department of the Medical Examiner, City and County of Honolulu, Honolulu, HI, USA

**Keywords:** Forensic taphonomy, Medicolegal death investigation, Season, Cadaver decomposition island, Tropics, Terrestrial ecology

## Abstract

Decomposition studies have been conducted in several regions of the world, but relatively few have investigated taphonomy in tropical environments. Even fewer have explored carcass decomposition during multiple tropical seasons, leaving the relationships between season and decomposition in tropical environments poorly understood. Ten decomposition studies using 30 carcasses were conducted in Honolulu, Hawaii, USA to start addressing this knowledge gap. These studies show that some postmortem processes were observed regardless of season. Carcass temperature and chemistry were spatiotemporally variable. Fly larval masses were consistently observed within 3 days (∼75 ADD) postmortem and carcasses lost 60%–90% of mass by 10 days (∼250 ADD) postmortem (Total Body Score ∼26). Season had a significant effect on decomposition, yet the warmest and most humid seasons did not always result in the most rapid and extensive decomposition. Seasonal variation appears to be less pronounced than at other tropical decomposition sites.

## 1 Introduction

Taphonomy is a term that refers to the ensemble of processes associated with the decomposition and preservation of organic materials [[1], [2], [3]]. Taphonomy is of substantial interest to several scientific disciplines because decomposition is a process that contributes to the cycling of energy and nutrients; decomposition is crucial to life on Earth. The taphonomy of animal remains, particularly mammal remains, receives significant interest because carcasses contribute to fundamental ecosystem functions and processes [4,5], including nutrient cycling [6,7], microbial community assembly [8,9], and scavenger ecology [10,11]. The ecological significance of decomposition can moreover have characteristics that are informative to criminal and medicolegal death investigations [12,13], the administration of justice [14] and the management of public health [15]. Decomposing remains have been studied on every continent [11,[16], [17], [18], [19], [20], [21]] and the understanding of carcass decomposition has advanced significantly since the turn of the 21st century. However, myriad variables that affect carcass breakdown still remain poorly understood. The purpose of the current work is to advance the understanding of decomposition in tropical habitats, defined here as terrestrial sites located between the Tropics of Cancer and Capricorn.

Fundamentally, carcasses are nutrient and moisture rich resource patches that attract several decomposers and release a rich concentration of chemicals into their environment [22,23]. Carcass decomposition can vary across climate and season such that remains tend to decompose at a greater rate in warmer, humid locations. This decomposition is often driven by insect [24] and microbial [25,26] activity. In contrast, decomposition tends to progress at a lesser rate in colder seasons, when insects are less successful against scavengers or overwintering [[27], [28], [29]]. The rates and patterns of decomposition are not constant over time [23]. The physical, chemical, and biological characteristics of a carcass change as moisture and nutrients are cycled throughout the landscape [22]. The need to better understand this spatiotemporal variation has prompted the need for decomposition studies and decomposition facilities around the world. In fact, human decomposition facilities have been established on three continents to better understand the influence of anthropogenic variables, such as clothing and burial, on the decomposition of human remains [30]. Decomposition facilities are thus sites that enable scientists to examine variation in taphonomy across space and time.

A large proportion of decomposition facilities are associated with forensic science in some way and are used to develop techniques for medicolegal death investigation such as evaluating the extent of decomposition of remains [31,32], estimating postmortem interval [28,33,34], and locating clandestine graves or dispersed remains [[35], [36], [37]]. The potential for forensic applications applies to the decomposition facility used in the current study, hereinafter referred to as the Chaminade facility. Studies conducted at the Chaminade facility attempt to consider synergistically both the forensic and ecological facets of decomposition. Decomposition studies conducted at the Chaminade facility are not necessarily focused on generating data relevant to the decomposition of adult human remains. The ultimate goal is to identify decomposition data that appear to have forensic value and then start collecting these data from decomposing human remains, whether they be juvenile or adult, in collaboration with medicolegal death investigation agencies.

Several decomposition studies have been conducted in the tropics, particularly in Brazil [38], Malaysia [39], Nigeria [40], and the USA – Hawaii [41]. These studies focused on a wide range of questions related to medicolegal death investigation [42], forensic microbiology [43], forensic entomology [44], scavenger ecology [45] cemetery planning [46], and public health [47]. In general, decomposition in the tropics tends to occur rapidly due to warm temperature, high humidity, and year-round insect activity. Yet one poorly understood aspect of tropical taphonomy is the variation in decomposition processes across seasons. Tropical locations can be associated with significant changes in temperature, precipitation, and humidity throughout the year [48]. Wang et al. [49] observed a significant decrease in the rate of pig (*Sus scrofa domesticus*) carcass decomposition during the cooler, less humid winter in Zhongsang City, Guangdong Province, China. Lira et al. [38] observed that the skeletonization of pig carcasses in the Brazilian Cerrado during the dry season (April–September) required nearly double the time to decompose relative to the wet season (October–March). Otherwise, tropical decomposition studies have been conducted to investigate the effect of other variables, such as placement in a built environment [39], burial [40], embalming [50], or clothing [51], without an analysis of seasonal influences.

The current work represents an initial attempt to establish a fundamental understanding of porcine carcass decomposition at the Chaminade facility. The primary goal of the current work is to characterize seasonal variation in carcass decomposition including the frequency, pattern, and timing of decomposition processes. The current work aims to serve as a foundation for future research that will continue to elucidate seasonality and carcass breakdown. The long-term goals of the decomposition studies conducted at the Chaminade faculty are to identify decomposition processes that can inform medicolegal death investigation and terrestrial ecology.

## 2 Materials and methods

Ten decomposition studies were conducted (Table 1) at the Chaminade site. Three domestic pig (*Sus scrofa domesticus*) carcasses were decomposed in each study and decomposition data were collected daily for 14 days in all but one study: the summer 2013 study was conducted for seven days. At least one decomposition study was conducted during each season, although most studies were conducted in the winter (n = 4) and summer (n = 3). A range of decomposition measurements were taken during these studies, but not every measurement was made during every study depending on the decomposition trajectories of the carcasses (Table S1). Individual decomposition studies conducted at this site have been published elsewhere [41,[52], [53], [54], [55]] and include detailed descriptions of materials and methods relevant to those studies.

**Table 1:** Ten decomposition studies were conducted at a tropical taphonomy facility in Honolulu, HI, USA (21° 17′ 27″ N, 157° 48’ 18” W) from 2013 to 2022. All but one study (summer 2013) was conducted for 14 days and at least one study per season was conducted. Three pig (*Sus scrofa domesticus*) carcasses were decomposed in each study. Carcasses were acquired from multiple farms on Oahu, which resulted in three causes of death. Carcass mass and sex data are presented in paired order. For example, the summer 2014 study included a 29 kg female (♀) and a 23 kg male (♂) pig carcass. Temperature and relative humidity data represent mean ± standard error.

Year	Dates	Season	Carcass Mass (kg)	Carcass Sex	Cause of Death	Mass Loss Measured	Temperature (°C)	Relative Humidity (%)
2013	24 June – 1 July	Summer	25, 28, 30	♀, ♀, ♀	Electrocution	No	27.0 ± 0.6	75.1 ± 3.9

2014	9–23 June	Summer	29, 23, 24	♀, ♂, ♀	Electrocution	Yes	26.3 ± 0.2	69.2 ± 1.2

2014	15–29 December	Winter	20, 23, 22	♀, ♀, ♀	Electrocution	Yes	23.6 ± 0.4	81.2 ± 2.0

2016	8–22 February	Winter	17, 16, 27	♂, ♀, ♂	Sharp force trauma	Yes	24.8 ± 0.4	66.9 ± 2.0

2017	28 February – 7 March	Winter	55, 58, 39	♂, ♀, ♂	Blunt force trauma	Yes	24.1 ± 0.4	88.3 + 2.2

2018	9–23 April	Spring	31, 36, 23	♀, ♀, ♀	Sharp force trauma	Yes	23.7 ± 0.3	86.3 ± 1.8

2019	6–20 March	Winter	29, 33, 34	♀, ♀, ♂	Sharp force trauma	No	24.4 ± 0.5	70.9 ± 2.6

2020	23 July – 6 August	Summer	33, 38, 35	♂, ♂, ♂	Sharp force trauma	No	26.1 ± 0.3	75.5 ± 1.9

2020	19 October – 2 November	Autumn	30, 26, 24	♀, ♂, ♀	Sharp force trauma	No	25.9 ± 0.2	87.3 ± 1.8

2022	30 March – 13 April	Spring	40, 39, 43	♂, ♂, ♀	Sharp force trauma	No	24.7 ± 0.2	72.3 ± 0.9

### 2.1 Site description & environmental characteristics

The decomposition site is located in a tropical savanna (Aw) climate [48] in Palolo Valley, Oahu, Hawaii (21° 17′ 27″ N, 157° 48’ 17” W). This site is approximately 87 m above sea level with mean annual precipitation of approximately 700 mm, 70% of which occurs in the autumn and winter (October–March). The site is rocky and its vegetation is dominated by guinea grass (*Megathyrsus maximus*) with night blooming cereus (*Hylocereus undatus*), aloe (*Aloe* spp.), and carrion plants (*Stapelia gigantia*) (Fig. S1). In terms of vertebrate scavengers, only the small Asian mongoose (*Herpestes javanicus*) has been observed at the facility [54]. The decomposition site is unfenced and all carcasses were uncaged due to minimal scavenging activity.

**Fig. 1:**
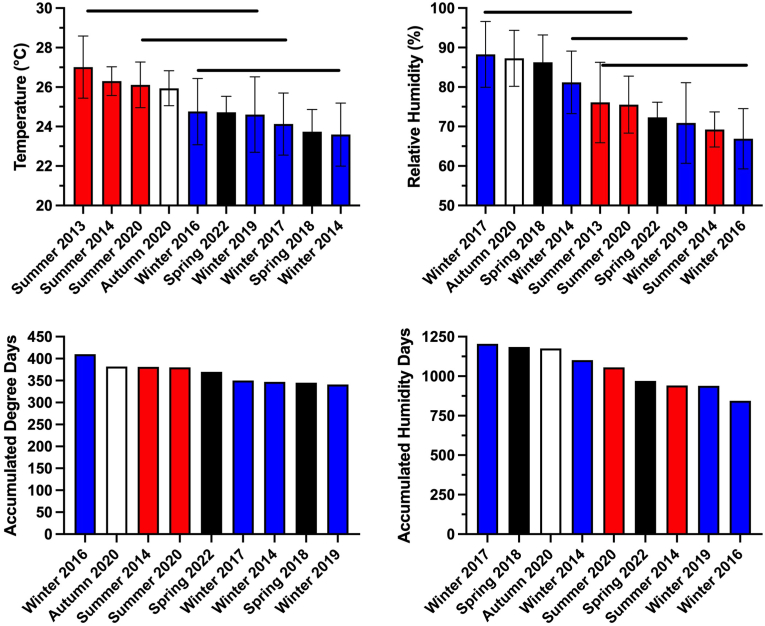
Average temperature (°C) and relative humidity (%) during the decomposition studies conducted at a tropical taphonomy facility located in Honolulu, HI, USA. Colors represent autumn (□), spring (), summer (), and winter () seasons. Horizontal bars represent statistical similarity: studies that share a bar are not significantly (p < 0.05) different. Vertical error bars represent standard errors where n = 336. Summer 2013 temperature data were not used to calculate Accumulated Degree Days because that study was 7 days in duration. All other studies were 14 days. (For interpretation of the references to color in this figure legend, the reader is referred to the Web version of this article.)

Ambient temperature (°C) and relative humidity (%) were measured at intervals of 1 h using a datalogger (HOBO U23 Pro v2, Product #U23-001, Onset Corp., Cape Cod, MA, USA) located at the site. Accumulated Degree Days (ADD) were calculated using 0 °C as the minimum developmental threshold. Accumulated Humidity Days (AHD) were calculated using 0% relative humidity as the threshold. See Fig. 1 for mean study temperature, relative humidity, ADD, and AHD. See Fig. S1 for mean daily temperature, relative humidity, ADD, and AHD.

### 2.2 Carcass characteristics

**Fig. 2:**
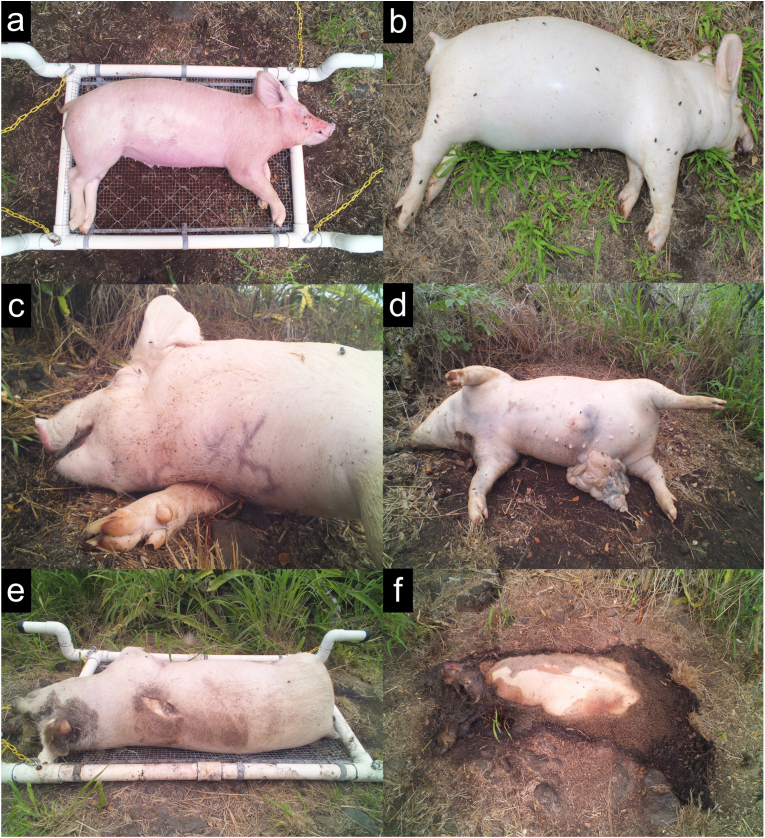
Commonly observed decomposition processes associated with pig (*Sus scrofa domesticus*) carcasses at a tropical taphonomy facility in Honolulu, HI, USA. All carcasses were placed at the facility in a fresh state of decomposition within 2 h of death (a). A total of 10 decomposition studies were conducted at the facility, five of which used a frame to weigh carcass mass (a, e). All carcasses were bloated (b) and presented several postmortem changes including marbling (c), abdominal rupture (d), and ruptures in other regions (e). Patent rigor mortis (d) was observed in 67% of carcasses and fly larval masses (f) were established in all carcasses between 3 and 10 days postmortem. Postmortem interval for each carcass: (a) 2.5 h, (b) 46 ADD/53 h, (c) 29 ADD/31 h, (d) 55 ADD/55 h, (e) 74 ADD/78 h, (f) 106 ADD/103 h.

Carcasses ranged in mass from 16 kg to 58 kg, with an average mass of 27 kg (Table 1). Carcass length equalled approximately 81 cm–94 cm and carcass height equalled approximately 58 cm–63 cm. The skin pigmentation of all pig carcasses was pink, with the exception of one which was black (winter 2017 study). All carcasses were acquired from local farms on Oahu, Hawaii. Carcasses were acquired from multiple farms due to practical reasons beyond our control. As a result, the cause of death and carcass mass varied across studies (Table 1). Although there was variation in cause of death, the protocols were consistent. Carcasses that underwent electrocution received the treatment behind one of their ears. Sharp force trauma was applied to the neck/chest (see Fig. 2d). A captive bolt gun was used to apply blunt force trauma to the forehead. Carcass acquisition, handling, transport, and placement were consistent throughout the ten studies; carcasses were collected within 15 min of death and placed at the decomposition site within 2 h of death. Carcasses designated for mass loss measurements were placed on a frame to allow the use of a hanging balance (See Fig. 2a and e). The distance between carcasses was not the same in all studies, but carcasses were always a minimum of 5 m apart. All carcasses were handled in accordance with the Institutional Animal Care and Use Committee Policy on Animal Material and State of Hawaii Statute §159–21.

### 2.3 Decomposition metrics – Total Body Score & mass loss

Three carcass decomposition metrics were collected daily. Total Body Score (TBS) was measured after Megyesi et al. [56] and Keough et al. [57]. Megyesi et al. [56] TBS was measured in all decomposition studies whilst Keough et al. [57] TBS was measured in six studies from winter 2017 through spring 2022. The latter method was designed to assess pig carcass decomposition whereas the Megyesi et al. [56] method from which it derives was developed for human decomposition. Carcass mass loss was measured in five studies from summer 2014 to spring 2018 (Table S1). Mass loss was measured by connecting a hanging balance to the weighing frame (see Fig. 2a and e) and lifting the frame with carcass off the ground [54]. This was not an automated process. It was conducted by members of the research team.

### 2.4 Skin metrics – temperature, pH, and oxidation reduction potential (Eh)

The first decomposition study to collect skin data was conducted in winter 2016. One meter was used to measure temperature and pH (Extech Instruments, #PH100, Nashua, NH, USA). A separate meter was used to measure oxidation reduction potential (Eh) (Extech Instruments, #RE300, Nashua, NH, USA). These measurements were collected daily from skin on the limbs, where the limb meets the trunk. This area was selected because skin in this area tended to persist through the initial 14 days of decomposition [52,54,55]. Measurements were taken from forelimbs and hindlimbs furthest from the ground. Measurements were collected from the same location on each carcass unless that area of skin decomposed, when these measurements were either collected from other remaining skin or stopped due to a lack of skin.

### 2.5 Larval mass metrics – temperature, pH, and oxidation reduction potential (Eh)

Larval mass data were collected during every decomposition study. The initial three decomposition studies (summer 2013, summer 2014, winter 2014) used a portable meter (Hach, Product #H170-G, Loveland, CO, USA) with sensors to measure temperature (ThermoWorks, Product #221–071, Lindon, Utah, USA), pH (Hach, Product #PHW77-SS, Loveland,CO, USA), and oxidation reduction potential (Eh) (Hach, Product #ORP110-GS, Loveland, CO, USA). Beginning in winter 2016, a portable meter was used to measure temperature and pH (Extech Instruments, #PH100, Nashua, NH, USA) with a separate meter to measure oxidation reduction potential (Eh) (Extech Instruments, #RE300, Nashua, NH, USA). These measurements were collected daily from conspicuous fly larval masses, which occurred between 3 days and 10 days postmortem. Sensors were inserted approximately 2.5 cm into the larval mass. Measurements were collected from the same area of the larval mass for as long as possible. Sensors then followed larval masses as they moved throughout the carcass.

### 2.6 Statistical analyses

All statistics and graphs were generated using Prism 9 for macOS (GraphPad Software, LLC, San Diego, CA, USA). Between-season comparison of decomposition (TBS, mass loss) and environmental (temperature, relative humidity, ADD) metrics were conducted using the Kruskall – Wallis test. The focus of the current study was placed on generating univariate statistics to advance the understanding of decomposition processes at the Chaminade facility. This focus will shift to the generation of multivariate statistics as more decomposition studies are conducted. This approach is described further in the Discussion.

## 3 Results

### 3.1 Ambient temperature and relative humidity

Mean hourly temperature (Fig. S1) ranged from 20 °C–30 °C. Decomposition study temperature was greatest in summer and autumn studies, although not always significantly greater than other seasons (Fig. 1). Study temperature did not predict maximum ADD although summer and autumn studies tended to reach greater maximum ADD. Mean hourly relative humidity (Fig. S1) ranged from 55%–100% and the seasonal effects on average relative humidity were unpredictable; the five most humid studies included at least one study from each season (Fig. 1). Average relative humidity ranged from 67%–88% with four studies (winter 2017, autumn 2020, spring 2018, winter 2014) averaging greater than 80% relative humidity.

### 3.2 Decomposition – frequency and timing of gross postmortem changes

Several postmortem processes were observed visually (Table 2, Fig. 2) and multiple processes were observed in greater than 70% of carcasses. Bloating, larval masses, and larval mass migration were observed in all carcasses. Marbling, skin slippage, abdominal rupture, and other ruptures were observed in 70%–94% of carcasses. Other ruptures were observed in the anus, back, and shoulder (Fig. 2e). Multiple other postmortem processes were observed less often. Patent rigor mortis (Fig. 2) and the disarticulation of limbs were observed in 60%–70% of carcasses. The exposure of ribs, disarticulation of the mandible, traces of scavenging, and total loss of skin were observed with less than 50% of carcasses.

**Table 2:**
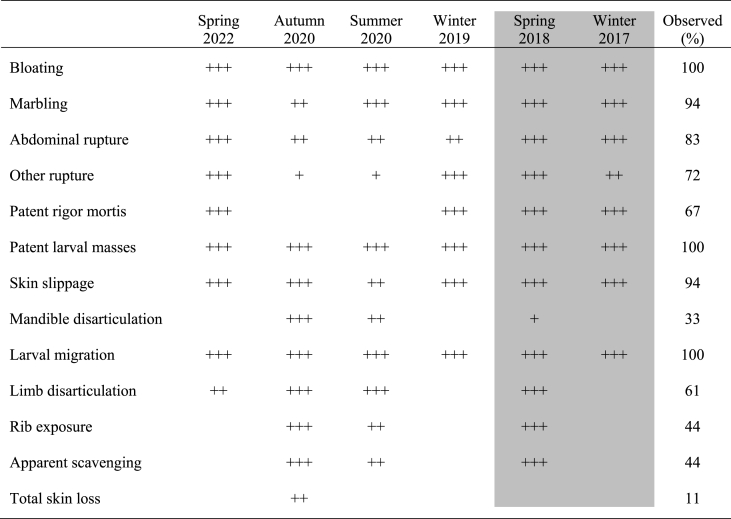
Frequency of decomposition processes where + indicates number of replicates, e.g., ++ represents two of three replicates. Column shading indicates a decomposition study that used frames to measure carcass mass. The presence of these frames might have affected the frequency of postmortem processes.

**Fig. 3:**
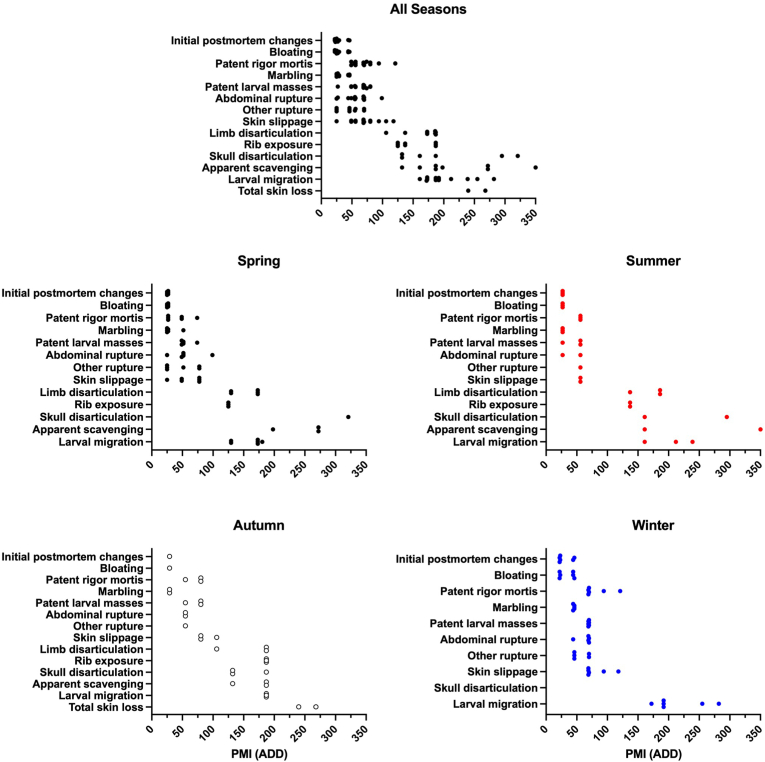
Initial occurrence of decomposition processes associated with pig (*Sus scrofa domesticus*) carcasses at a tropical taphonomy facility in Honolulu, HI, USA. Many processes (bloating, rigor mortis, marbling, larval masses, rupture) were initially observed prior to ∼120 ADD (4 days) while other processes tended to occur between ∼100 ADD – 350 ADD (4–14 days). Missing data points are because many decomposition processes were not observed in 100% of carcasses (Table 1). Seasonal variation was also observed where decomposition processes were observed earliest in summer studies and latest in winter studies.

Postmortem processes were documented temporally and Fig. 3 indicates the PMI at which each one was initially observed. Eight processes were observed prior to 120 ADD; bloating, patent rigor mortis, marbling, patent larval masses, abdominal rupture, other rupture, and skin slippage. The remaining processes were observed from approximately 100 ADD – 350 ADD; limb disarticulation, rib exposure, mandible disarticulation, apparent scavenging, larval migration, and total skin loss. These groups of postmortem processes will hereinafter be referred to as the ‘Early group’ and ‘Late group’ for comparison purposes.

Some seasonal variation in the timing of postmortem processes was observed. All Early group processes in spring, summer, autumn, and winter were observed prior to 100 ADD, 60 ADD, 110 ADD, and 120 ADD, respectively. All Late group processes were observed following 120 ADD, 120 ADD, 100 ADD, and 150 ADD, respectively. The timing of larval migration was of particular interest because the completion of this process is used as an indicator of the Advanced Decay stage of decomposition [23]. The timing of larval migration also varied by season and ranged from 125 ADD – 175 ADD, 150 ADD – 250 ADD, 175 ADD – 200 ADD, and 175 ADD – 275 ADD, in spring, summer, autumn, and winter, respectively.

### 3.3 Decomposition metrics – Total Body Score & mass loss

**Fig. 4:**
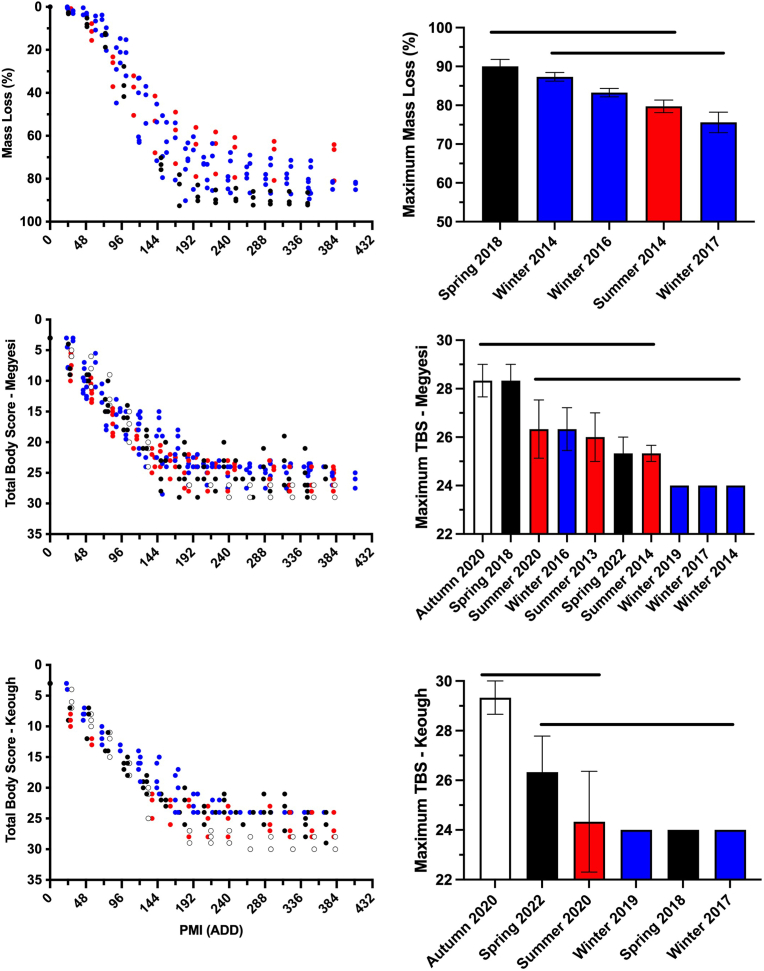
Decomposition metrics of pig (*Sus scrofa domesticus*) carcasses at a tropical taphonomy facility in Honolulu, HI, USA. Carcass breakdown was rapid regardless of season. Colors represent autumn (○), spring (), summer (), and winter () decomposition studies. Horizontal bars represent statistical similarity: studies that share a bar are not significantly (p < 0.05) different. Vertical error bars represent standard errors where n = 3. (For interpretation of the references to color in this figure legend, the reader is referred to the Web version of this article.)

Carcass decomposition progressed rapidly, regardless of season and decomposition metric. Both TBS methods yielded similar decomposition patterns (Fig. 4). However, TBS differed from mass loss data because TBS was not associated with an initial lag period. Instead, TBS increased within 25 ADD (24 h) and continued doing so until approximately 200 ADD (8 days). Maximum TBS in all studies ranged from 24 to 29 and these were reached by approximately 270 ADD (10 days).

Little mass was lost prior to 50 ADD (2 days) (Fig. 4). However, carcasses lost approximately 80% of mass by ∼230 ADD (9 days). This was not affected by carcass color [53]. Rapid decomposition coincided with the presence of patent fly larval masses throughout the remains and mass loss slowed following their migration by 200 ADD – 300 ADD. By this time the remains presented brown and black discoloration of the skin, some bone exposure, and patent decomposition fluids in associated soil. Decomposition fluids at this time were viscous and very dark in color. They contained other materials including hair, dead fly larvae, dead millipedes, dead land snails, soil aggregates, and a variety of plant materials like leaves and stems. These characteristics represent the Advanced Decay stage of decomposition [see 23].

Significant (p ≤ 0.05) seasonal differences were observed with all decomposition metrics but these did not follow a clear pattern. For example, mass loss during spring 2018 was significantly (p ≤ 0.05) greater than in winter 2017 (Fig. 4) even though temperature and relative humidity were similar (Fig. 1). However, significant differences in mass loss were not observed between spring 2018, winter 2014, winter 2016, and summer 2014. The greatest maximum TBS was observed in autumn 2020, spring 2018, and spring 2022 but these were not significantly greater than multiple other studies.

### 3.4 Skin metrics – temperature, pH, and oxidation reduction potential (Eh)

**Fig. 5:**
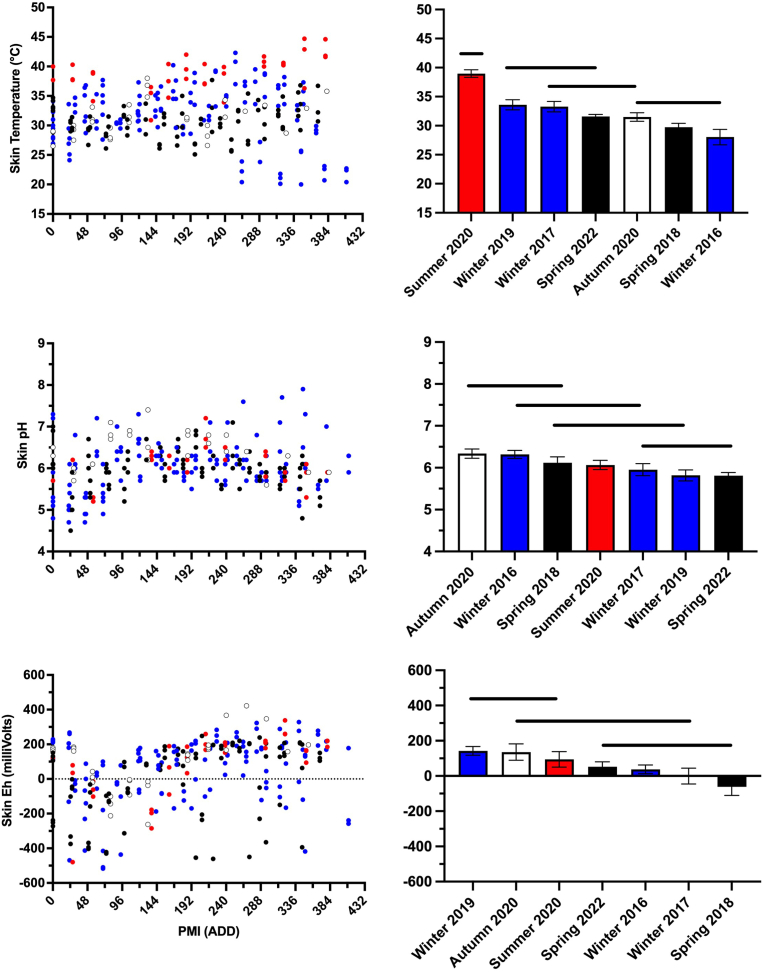
Skin temperature, pH, and oxidation reduction potential (Eh) of decomposing pig (*Susscrofa domesticus*) carcasses at a tropical taphonomy facility in Honolulu, HI, USA varied over time and between seasons. Colors represent autumn (○), spring (), summer (), and winter () decomposition studies. Horizontal bars represent statistical similarity: studies that share a bar are not significantly (p < 0.05) different. Vertical error bars represent standard errors where n = 45. (For interpretation of the references to color in this figure legend, the reader is referred to the Web version of this article.)

Skin temperature, pH, and Eh changed over time and differences between seasons were also observed (Fig. 5, Fig. S4**)**. Daily skin temperature ranged from 20 °C–45 °C. Mean summer 2020 skin temperature was significantly greater than all other studies, but most significant seasonal differences did not follow a consistent pattern (Fig. 5). However, a relatively consistent site temperature pattern was observed where ambient < skin < larval mass (Fig. S5). These differences were not always significant.

Daily skin pH values ranged from 4.5 to 7.9 while average skin pH ranged from 5.8 (spring 2022) to 6.3 (autumn 2020) (Fig. 5, Fig. S4). Significant seasonal differences were observed, although the average range equaled 0.5 pH values (Fig. 5). Like skin temperature, these differences were unpredictable and not defined clearly by season. Unpredictable seasonal differences were also observed in skin Eh. Average Eh ranged from approximately −60 mV (autumn 2020) to +140 mV (winter 2019). Daily Eh values were more variable, ranging from −500 mV to +400 mV (Fig. 5, Fig. S5).

### 3.5 Larval mass metrics – temperature, pH, and oxidation reduction potential (Eh)

**Fig. 6:**
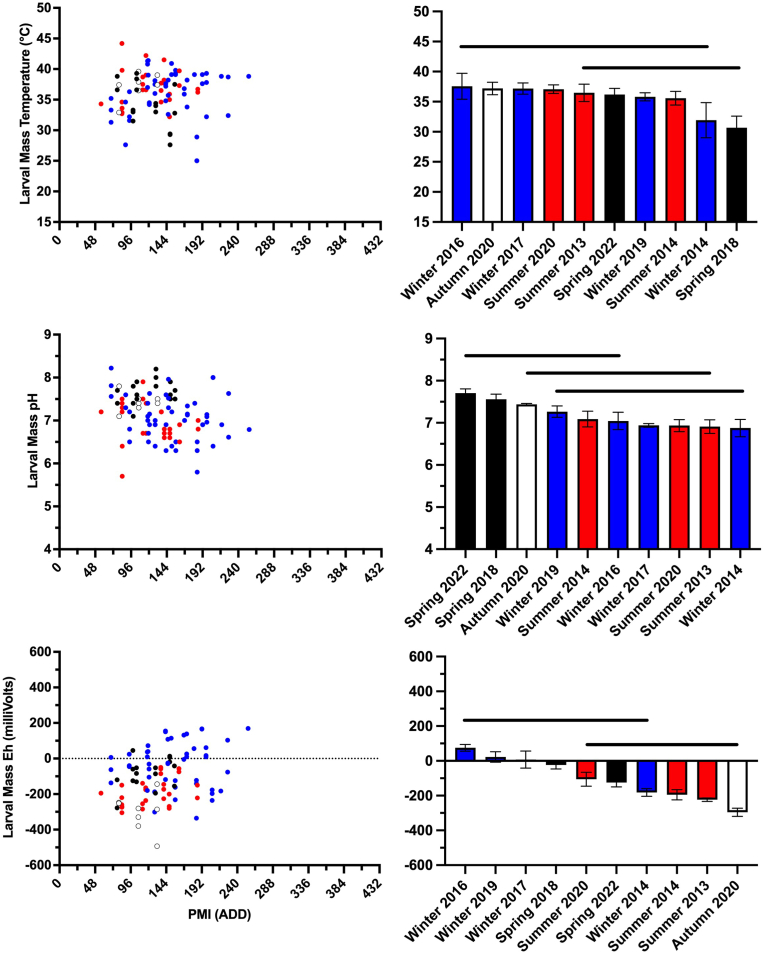
Fly larval mass temperature, pH, and oxidation reduction potential (Eh) of decomposing pig (*Sus scrofa domesticus*) carcasses at a tropical taphonomy facility in Honolulu, HI, USA varied over time and between seasons. Colors represent autumn (○), spring (), summer (), and winter () decomposition studies. Horizontal bars represent statistical similarity: studies that share a bar are not significantly (p < 0.05) different. Vertical error bars represent standard errors where n is variable, ranging from 6 to 18. (For interpretation of the references to color in this figure legend, the reader is referred to the Web version of this article.)

Larval mass temperature, pH, and Eh changed over time and differences between seasons were also observed. Larval mass measurements were always collected between 56 ADD and 255 ADD (3–10 days postmortem) (Fig. 6, Fig. S4) although the establishment and duration of larval masses varied between studies (Fig. S5**)**. Daily larval mass temperature ranged from 25 °C–44 °C while the range of average larval mass temperatures only ranged from 31 °C (spring 2018) to 38 °C (winter 2016).

Daily larval mass pH ranged from slightly acidic to slightly alkaline, 5.7–8.2, while average pH ranged from 6.9 (winter 2014) to 7.7 (spring 2022) (Fig. 6, Fig. S6). Like temperature, larval mass pH was consistently greater than, or equal to, skin pH (Fig. S6). Significant differences were observed between studies along with a seasonal effect, where greater pH values were observed in the spring and autumn studies.

Daily larval mass Eh ranged from −493 mV to +169 mV while average Eh ranged from −296 mV (autumn 2020) to +75 mV (winter 2016) (Fig. 6, Fig. S7). Differences were observed between skin Eh and larval mass Eh (Fig. S7) but they were not as marked as with temperature and pH. Larval mass Eh was usually less than, or equal to, skin Eh (Fig. S7). Significant differences were observed between seasons and a weak seasonal effect was observed: winter and spring studies tended to have greater Eh than summer and autumn studies (Fig. 6).

## 4 Discussion

The study of decomposition at the Chaminade facility provided an opportunity to better understand taphonomy at the site, including the frequency and timeline of postmortem processes in different seasons. All carcasses were colonized by fly larvae within 3 days (∼75 ADD) postmortem and proceeded to an advanced stage of decay by 10 days (∼250 ADD) postmortem. This corresponded to a 60%–90% loss of initial mass and a TBS of approximately 26. Carcass decomposition at the Chaminade facility was characterized by bloating, conspicuous fly larval masses, and larval mass migration regardless of season (Table 2). Fly larvae, which were responsible for the majority of soft tissue decomposition, consistently migrated from the remains within 10 days postmortem. Other decomposition processes, such as the disarticulation of the mandible and exposure of ribs, were observed in less than 50% of carcasses. The current data are foundational to understanding the sequence of taphonomy at this site — and in tropical environments — and serve to improve the general understanding of decomposition processes.

### 4.1 Rates of decomposition, mass loss, and link with seasonality

Season had a significant effect on decomposition. While some postmortem processes occurred consistently regardless of season, a clear and robust understanding of the relationship between taphonomy and season has yet to be achieved. For example, the greatest mass loss and gross postmortem change did not always occur during the warmer, more humid studies. Future work will continue to explore the relationship between season and postmortem processes by conducting more studies during the autumn and spring. Future work should also investigate the effects of initial carcass mass, cause of death, and use of a weighing frame (human intervention). These could be influential variables and an understanding of their effects will be prioritized as the dataset grows and is subject to multivariate analyses.

Two important observations were made when comparing the data from the Chaminade facility to other decomposition studies conducted in the tropics. First, carcass decomposition can vary greatly throughout the tropics. Second, the methods to measure carcass decomposition also vary greatly. We used mass loss and TBS as the primary metrics of decomposition while using decomposition stages based on Carter et al. [23] as a descriptor to complement these metrics. While several other studies also used mass loss or TBS, most of them relied upon qualitative decomposition stages as the primary decomposition metric. One interesting observation is that seasonal variation at the Chaminade facility was less pronounced than at other sites. Wang et al. [49] measured decomposition of pig carcasses (32 kg–67 kg) in a grassy woodland in Zhongsang City, Guongdong Province, China and observed a similar rate of decomposition during spring, summer, and autumn: carcasses reached an advanced stage of decay in 8–10 days postmortem. However, 49 days were required to reach an equivalent stage during the winter when mean ambient temperature and relative humidity equaled 18.5 °C and 62%, respectively. A relatively similar observation was observed by Lira et al. [38] in a woodland and a savanna in the Cerrado, eastern Brazil, during the rainy season (October–March) and dry season (April–September). Smaller (2.0 kg–2.5 kg) pig carcasses reached an advanced stage of decomposition in 8 days during the rainy season and 14 days during the dry season. Other tropical decomposition studies, although not seasonal in nature, reported rates of pig carcass breakdown that are comparable to the present findings. These studies were conducted in a forest in Shenzhen City, Guangdong, China [58], forest-savanna near Okuku, Cross River State, Nigeria [20,59,60], Ekwulumini, Anambra State, Nigeria [61], and Medellín, Colombia [62,63]. These studies show that the decomposition data generated at the Chaminade facility can be representative of decomposition processes in other parts of the tropics.

While not season focused, mass loss data from other pig decomposition studies conducted throughout the State of Hawaii are roughly similar to the mass loss of carcasses at the Chaminade facility. The decomposition of 10 kg–25 kg pig carcasses in Diamond Head (volcanic tuff cone), Oahu [64,65] during summer/autumn was similar to the current study, as decomposition was driven by insect activity and resulted in a loss of 70%–80% of carcass mass by 10 days postmortem. This similarity is not surprising considering that Diamond Head and the Chaminade facility are analogous habitats, but similar decomposition processes were also observed in contrasting areas in Hawaii. The mass loss of 10 kg–16 kg pig carcasses in a rainforest at Lyon Arboretum, Oahu during spring/summer presented a similar rate and extent of decomposition [64,66]. Furthermore, decomposition in a rainforest and lava field on the island of Hawaii, approximately 500 km from the Chaminade facility, during spring/early summer was also driven by insect activity and resulted in a loss of 70%–80% of carcass mass by 10 days postmortem [67].

To some extent decomposition at the Chaminade facility also differed from other studies in the tropics, including Hawaii. Pig carcasses (8 kg–15 kg) in Diamond Head, Oahu [68] during the summer lost a similar percentage of mass but required approximately 5 more days to do so. Pig carcasses (antemortem mass not reported) on Coconut Island and Barber's Point, Oahu, Hawaii during winter/early spring followed a similar timeline as the Chaminade carcasses, but some of them lost approximately 10% more total mass [69]. The Chaminade facility data also differed from some tropical decomposition studies conducted at higher elevations (1800+ m) such as Mauna Loa, Hawaii [67], Los Altos de Chiapas, Mexico [70], Paramo, Chingaza National Park, Colombia [71], and Usaquén, Bogotá, Colombia [72] where decomposition was slower by a number of days. However, greater elevation alone did not indicate a slower rate of decomposition, as decomposition studies conducted at 1169 m [67] and 1450 m [62,63] reported results similar to the current study.

Although we consider the rate of decomposition at the Chaminade facility to be rapid, there are data to show that carcasses can reach an advanced stage of breakdown within 5–8 days postmortem. These studies were conducted in a shrubland near Serra Talhada, Pernambuco State, Brazil using 15 kg pig carcasses [73] and in a forest near Tuxtla Gutierrez, Chiapas, Mexico using 9 kg–12 kg pig carcasses [74]. However, the latter study was conducted with clothed carcasses that might have affected decomposition [[75], [76], [77]].

These rapid rates of decomposition are not unique to tropical habitats. Spicka et al. [78] observed similar mass loss and gross postmortem change of 20 kg–50 kg pig carcasses in a temperate habitat near Lincoln, Nebraska, USA during the summer. These similarities might indicate the possibility for insightful comparisons across climates where decomposition studies conducted in one region might inform investigations elsewhere regardless of climate type, latitude, longitude, and elevation. This coordination of data would be particularly helpful to regions of the world where experimental decomposition studies under controlled conditions are not possible.

One important observation to recognize when comparing the data from the Chaminade facility to other studies is the frequency at which the index of decomposition (mass loss, TBS, stages) affected the interpretation of the present findings. For example, the mass loss of pig carcasses in a rainforest, on a lava field [67], and in a grassland [65] in Hawaii was similar to the mass loss observed at the Chaminade facility. However, the decomposition stages ascribed to these carcasses lagged behind the stages at the Chaminade facility by several days. A similar phenomenon was observed in studies from Nigeria [79,80] where TBS in Okuku, Cross River State and Ekwulumini, Anambra State were similar to the TBS from the Chaminade facility. In contrast, the decomposition stages at these Nigerian sites occurred several days after the stages observed at the Chaminade facility. These differences in the timeline of decomposition stages were characterized by longer Active Decay. Although decomposition stages, TBS, and mass loss are meant to measure decomposition and resource change, they apparently capture different aspects of these processes. Dawson et al. [19] observed that a continuous measure of resource change, such as TBS and mass loss, provided greater resolution to taphonomic analysis. They [19] also found that TBS was the key driver of insect species richness and community composition on pig and human remains. Decomposition stages are certainly helpful to facilitate the discussion of taphonomy but they probably should not be the sole measure of decomposition. These observations support the Finaughty et al. [81] recommendation for using quantitative measures to optimize the precision of taphonomy data.

### 4.2 Insights into the Cadaver Decomposition Island (CDI)

**Fig. 7:**
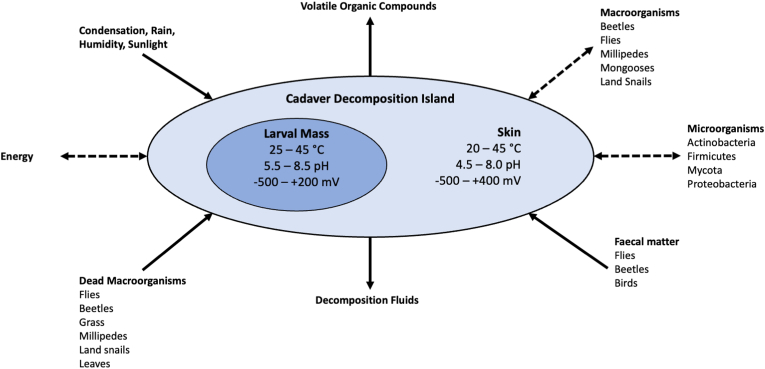
Cadaver Decomposition Island (CDI) characteristics of decomposing pig (*Sus scrofa domesticus*) carcasses at a tropical taphonomy facility in Honolulu, HI, USA. CDIs were a hub of activity and source of decomposition products that varied spatially and temporally. All carcasses (n = 30) decomposed rapidly, losing approximately 80% of mass by ∼230 ADD (9 days). Fly larval masses were essentially islands within each CDI presenting temperature, pH, and oxidation reduction potential that differed from the skin. Larval masses were established between 56 ADD - 255 ADD (3–10 days postmortem) regardless of season. Figure adapted from Carter et al. [4].

The current data also provide some resolution to the biophysicochemical characteristics of CDIs. Each pig carcass was large enough to sustain significant biological and chemical activity (Fig. 7, Fig. S8). Measurements of skin and larval masses demonstrated the spatiotemporal heterogeneity of carcass temperature, pH, and Eh (Fig. 7). Larval masses were patches within a CDI that tended to be warmer and less acidic than the skin while the Eh of larval masses was similar to, or slightly more reducing, than the skin. In a complementary study, the bacterial community cultured from larval masses comprised fewer species than the community cultured from the skin [52] but the structure of both communities changed over time as the carcass decomposed. Junkins et al. [55] observed a significant inverse relationship between microbial community richness and Eh, where richness decreased over time. These microbes likely played a vital role in the release of VOCs detected by Dubois et al. [41] and decomposition fluids into the CDI.

Carcasses contributed to microbial, insect, mongoose, and plant communities. It would be beneficial to explore these trophic interactions in more detail, including to learn how carcass mass influences characteristics of the CDI. Carcass mass and body mass index are poorly understood variables associated with apparently contrasting experimental results [31,78,82,83]. Mason et al. [84] and Tarone et al. [85] observed relationships between human body mass index and microbial diversity while Dawson et al. (2021) observed little effect of carcass mass on insect communities. Insects are clearly a primary decomposer group in the tropics and it would be informative to know if there exists a threshold carcass mass below which there is a significant difference in decomposer community structure [78,[85], [86], [87]]. A mass threshold might indicate a different series of decomposition processes for smaller carcasses that affects the heterogeneity of the CDI by providing insufficient area for the formation of larval masses. Carcass mass is also highly relevant to medicolegal death investigation as human decedents of all sizes can undergo decomposition. Ultimately, the consideration of CDIs and their role in the function of terrestrial ecosystems [5,88,89] is crucial to the development of forensic taphonomy as a discipline, and it is hoped that the current observations will help stimulate continued research into carrion ecology [22,[90], [91], [92]].

### 4.3 Developing a regional taphonomy database for medicolegal death investigation

As a result of collaboration with several medicolegal agencies across the USA, it has not escaped our notice that the approaches used over the course of ten carcass decomposition studies have potential to be used in the context of actual medicolegal death investigations. Although pig and human remains can present different postmortem processes and resource changes [7,19,87,88,[93], [94], [95], [96], [97], [98]], some fundamental decomposition processes can be sufficiently similar to inform investigative decision-making where postmortem changes obscure or mimic criminal activity [13,98,99]. This does not mean that the current data are appropriate for direct application to actual medicolegal death investigations. Medicolegal decomposition cases with a known PMI can be used to develop a database for human remains in forensic scenarios. Retrospective and real-time decomposition studies conducted in collaboration with medicolegal death investigation agencies provide valuable insight into the taphonomy within a given jurisdiction, particularly in built environments [14,42,43,56,[100], [101], [102], [103], [104], [105], [106], [107]]. These data will likely provide investigators with an improved ability to determine if the extent of decomposition is consistent with the scene findings.

Similarly, we conclude that the synthesis of decomposition studies is an approach that is particularly valuable to human decomposition facilities. One of the greatest challenges for human decomposition facilities is to navigate the vast inherent variation between the donors including height, weight, cause of death, health at death, antemortem medical treatments, and time of death [108]. This variation can introduce uncertainty in the reconstruction of the decomposition processes and the estimation of the postmortem interval. One approach to capturing and managing intra- and inter-donor variation is to develop a long-term taphonomy database that will eventually elucidate the effects of these variables. We hope that the present study contributes to achieving that goal.

### 4.4 Study limitations and avenues for further research

The research presented here is unique in its large collection and synthesis of tropical taphonomic data. It is hoped that it will contribute in some way to the continued development of forensic taphonomy in tropical and other environments. Study limitations must however be addressed prior to any forensic application. The carcasses used across the years and seasons were imperfect replicates due to their differences in mass, cause of death, rearing regimen, and presence of a weighing frame. Also, the seasonal distribution of studies is weighted toward winter (n = 4) and summer (n = 3). These factors introduced variability which likely obscured some temporal patterns and may have decreased statistical power [109]. Follow-up studies could minimize these limitations by purchasing carcasses from the same farm and by requesting a specific carcass mass. It would also be informative to assess the effect, if any, of the weighing frame on decomposition processes. Carcass type, inter-carcass placement distances, sampling frequency, decomposition scoring methods, and probes would however likely remain the same to facilitate comparisons and encourage standardization [see 81].

Another potential source of variation is the placement of multiple carcasses for each study. It would be informative to study the placement of a single carcass to evaluate the site's baseline ecological response to carrion and to help identify potential multi-carcass effects [81]. This variable is particularly important for assessing the predictive power of the data and incorporating taphonomic research into medicolegal death investigation [110]. Future studies can moreover implement clothing or alternate locations to respectively investigate forensically relevant scenarios and to help capture the biogeographic variability of Oahu [81,111]. Managing limitations and identifying avenues for further research will be aided by the incorporation of multivariate statistical analysis and emerging theory [22, 81, [111], [112], [113]]. The current data have great potential for multivariate analyses and these will be prioritized as a more equal distribution of seasons is achieved. It is expected that multivariate analyses will reveal greater insight into the variables responsible for driving decomposition while univariate analyses will continue to identify fundamental trends.

## 5 Conclusions

In conclusion, the synthesis of ten carcass decomposition studies advanced the understanding of the timing and frequency of common postmortem processes at a tropical decomposition facility. Carcasses consistently decomposed rapidly and supported fly larval masses from 3 days (∼75 ADD) to 10 days (∼250 ADD) postmortem. Carcasses lost 60%–90% of initial mass and TBS equaled approximately 26 by the time of larval migration. Significant bloating was observed in all carcasses but several postmortem processes such as rupture, mandible disarticulation, and rib exposure occurred less frequently. Chemical analyses showed that a carcass is a heterogeneous resource patch characterized by spatiotemporal variation in temperature, pH, and Eh. The timing and extent of these decomposition processes were significantly influenced by season but this effect was not clear – the warmest, most humid seasons did not always correspond to the most rapid and extensive rates of breakdown. The current work also provides informative context to taphonomy by showing that seasonal variation at the Chaminade facility was less pronounced than other tropical locations. Ultimately, these and future data will be used to develop concepts and techniques suitable for medicolegal death investigations.

## CRediT authorship contribution statement

**David O. Carter:** Project administration, Supervision, Conceptualization, Formal analysis, Investigation, Methodology, Visualization, Writing – original draft, Writing – review & editing. **Adam Orimoto:** Investigation, Methodology, Writing – review & editing. **Carlos A. Gutierrez:** Investigation, Methodology, Writing – original draft, Writing – review & editing. **Agathe Ribéreau-Gayon:** Investigation, Methodology, Visualization, Writing – original draft, Writing – review & editing. **Emily L. Pecsi:** Investigation, Methodology, Visualization, Writing – original draft, Writing – review & editing. **Katelynn A. Perrault:** Writing – original draft, Writing – review & editing. **Alexis J.L. Peterson:** Conceptualization, Investigation, Methodology, Visualization, Writing – review & editing.

## Declaration of competing interest

The authors declare that they have no known competing financial interests or personal relationships that could have appeared to influence the work reported in this paper.
