# Supplementary material for: A synthesis of carcass decomposition studies conducted at a tropical (Aw) taphonomy facility: 2013–2022
Source: Forensic Sci Int Synerg. 2023 Aug 8;7:100345. doi: 10.1016/j.fsisyn.2023.100345 (PMC10440585; doi:10.1016/j.fsisyn.2023.100345)
Supplement: Multimedia component 1 [file mmc1.pdf]

Table S1. Summary of data collected during each decomposition study conducted at a tropical taphonomy facility in Honolulu, HI, USA. The presence of a black bar indicates that the measurement was collected. Only some data were collected during every decomposition study: ambient temperature & relative humidity, Total Body Score – Megyesi, and the temperature, pH, and oxidation-reduction potential (Eh) of insect larval masses. Other measurements were developed later and incorporated into the taphonomy workflow when possible. It is anticipated that additional measurements will be incorporated in the future while some measurements will eventually be eliminated.

	Summer 2013	Summer 2014	Winter 2014	Winter 2016	Winter 2017	Spring 2018	Winter 2019	Summer 2020	Autumn 2020	Spring 2022
Ambient Temperature & Relative Humidity	■	■	■	■	■	■	■	■	■	■
Total Body Score – Megyesi	■	■	■	■	■	■	■	■	■	■
Larval Mass Temperature	■	■	■	■	■	■	■	■	■	■
Larval Mass pH	■	■	■	■	■	■	■	■	■	■
Larval Mass Eh	■	■	■	■	■	■	■	■	■	■
Carcass Mass Loss		■	■	■	■	■				
Skin Temperature				■	■	■	■	■	■	■
Skin pH				■	■	■	■	■	■	■
Skin Eh				■	■	■	■	■	■	■
Total Body Score – Keough					■	■	■	■	■	■
Standardized Scene Photography					■	■	■	■	■	■
Frequency & Timing of Postmortem Processes					■	■	■	■	■	■

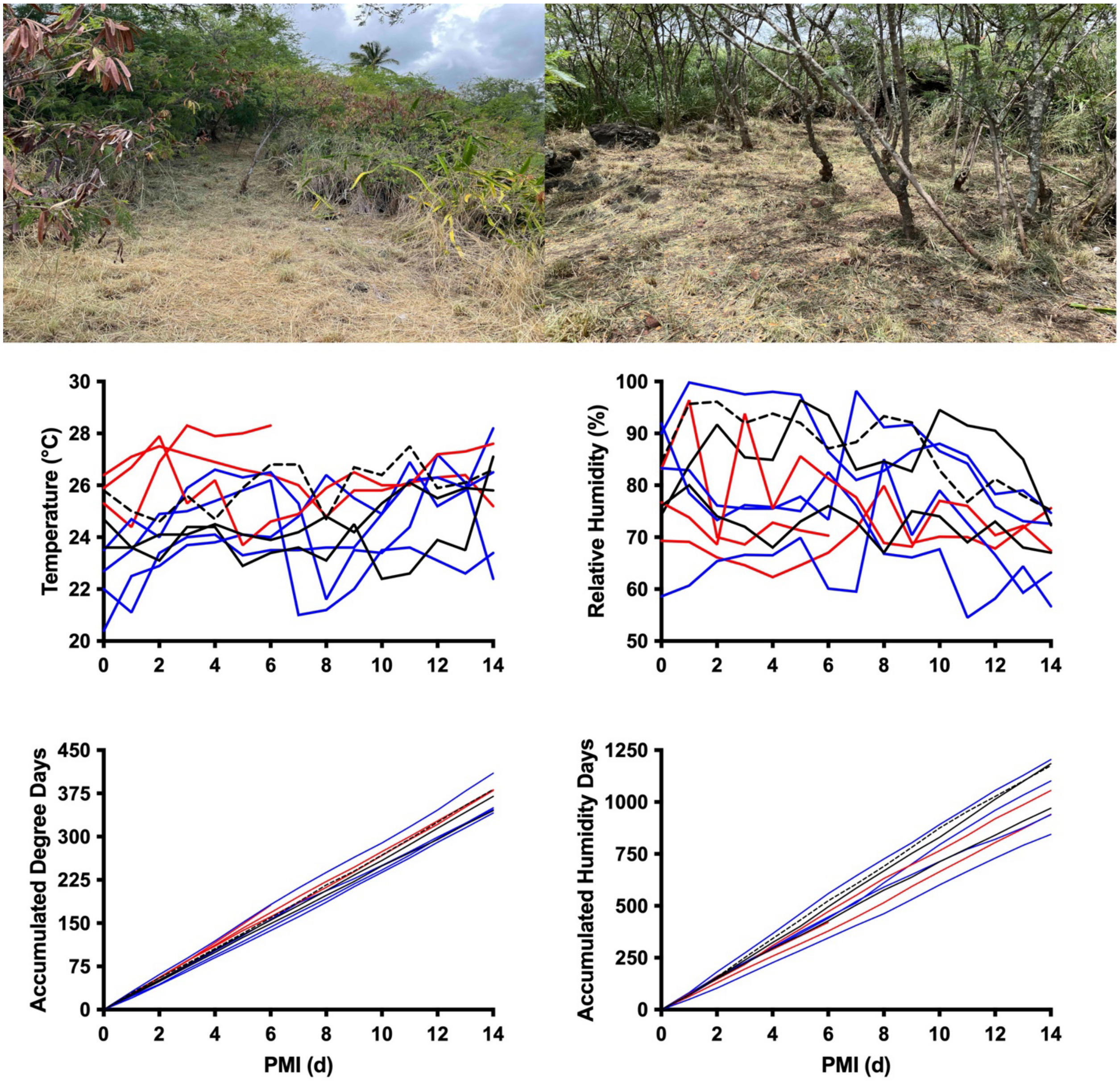

Figure S1. Site views of the tropical taphonomy facility at Chaminade University of Honolulu, Hawaii, USA during spring 2023 and hourly measurements of temperature (°C) and relative humidity (%) during the decomposition studies conducted in autumn (– –), spring (—), summer (—), and winter (—). Accumulated degree days were calculated as (maximum temperature + minimum temperature) ÷ 2 for each 24-hour period. Similarly, Accumulated Humidity Days were calculated as (maximum relative humidity + minimum relative humidity) ÷ 2 for each 24-hour period.

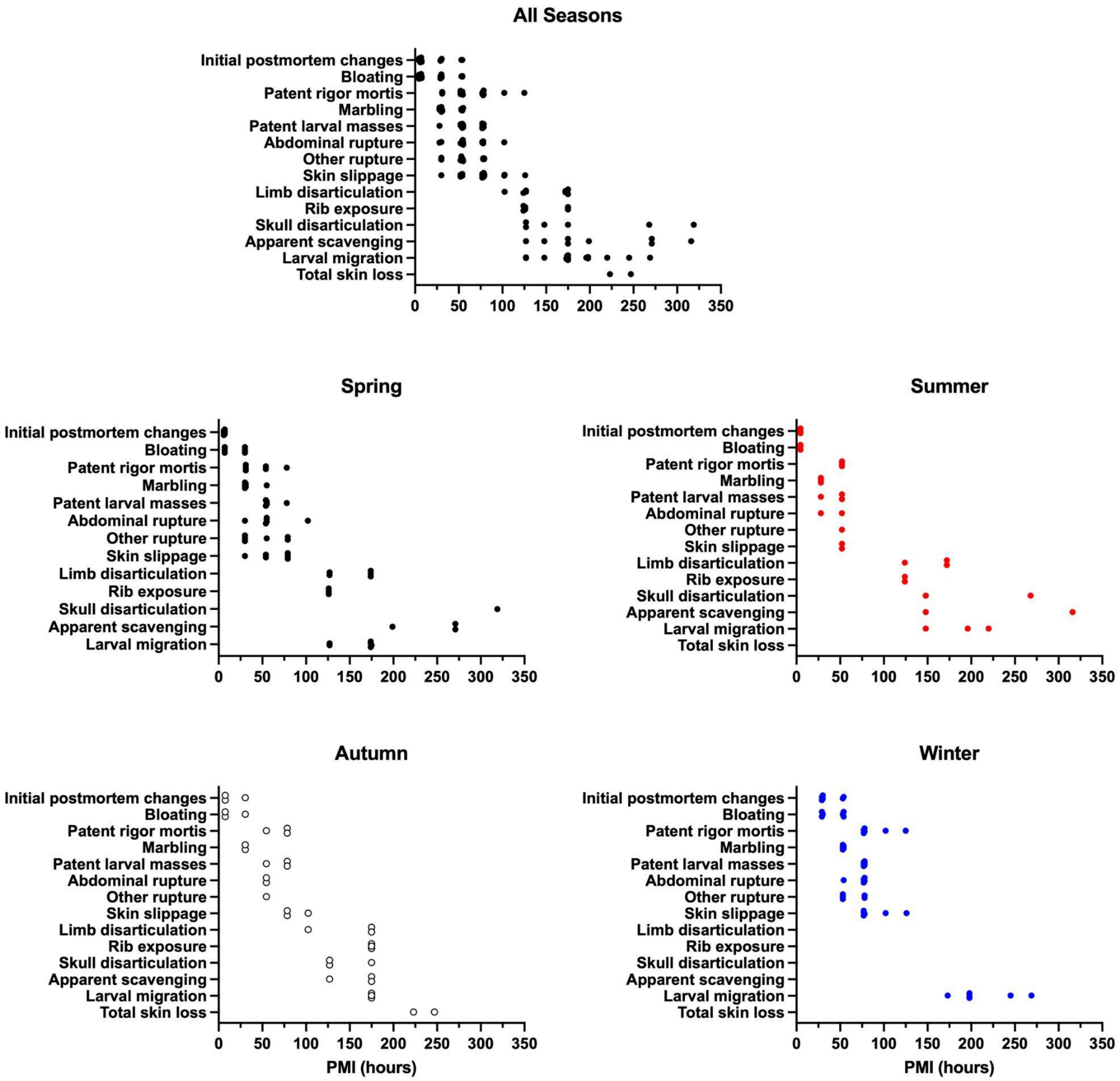

Figure S2. Decomposition of pig (*Sus scrofa domesticus*) carcasses at the Chaminade facility in Honolulu, Hawaii, USA were associated with several common decomposition processes and their initial occurrence was recorded. Many processes (bloating, rigor mortis, marbling, larval masses, rupture) were initially observed prior to ~100 hours postmortem while other processes tended to occur between ~100 hours - 325 hours. Missing data points are because most decomposition processes were not observed in 100% of carcasses (Table 1). Seasonal variation was also observed where decomposition processes were observed earliest in summer studies and latest in winter studies.

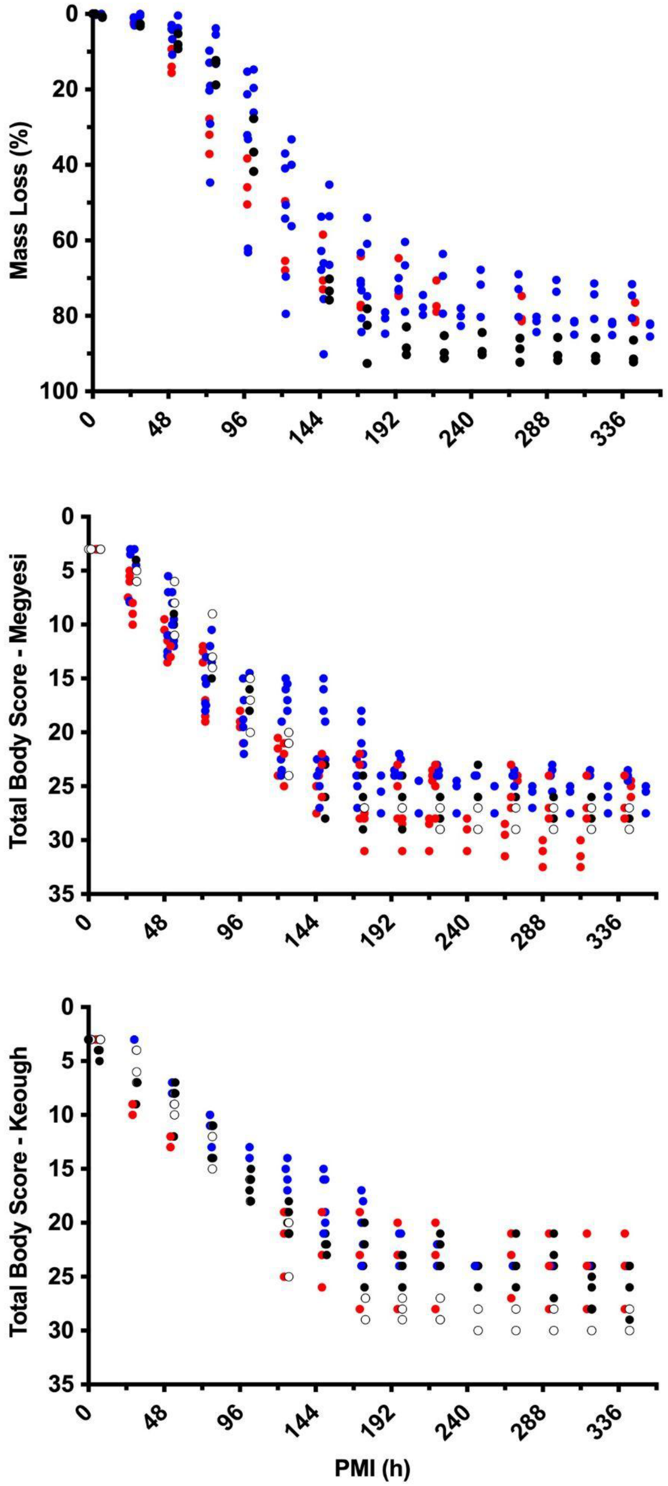

Figure S3. Decomposition of pig (*Sus scrofa domesticus*) carcasses at the Chaminade facility in Honolulu, Hawaii, USA was rapid regardless of season. Colors represent autumn (○), spring (●), summer (●), and winter (●) decomposition studies.

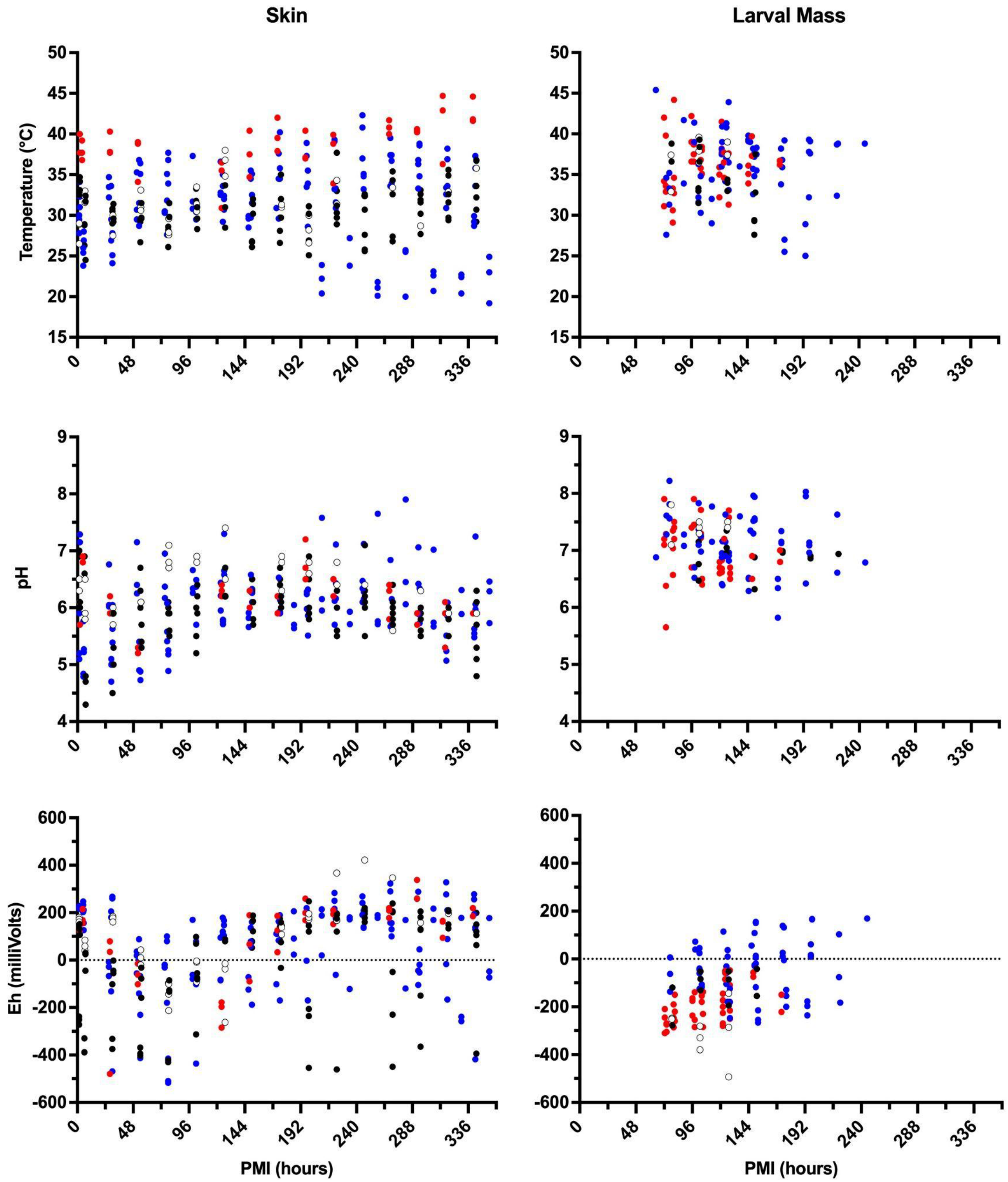

Figure S4. Skin temperature, pH, and oxidation reduction potential (Eh) of decomposing pig (*Sus scrofa domesticus*) carcasses at the Chaminade facility in Honolulu, Hawaii, USA varied over time and between seasons. Colors represent autumn (○), spring (●), summer (●), and winter (●) decomposition studies.

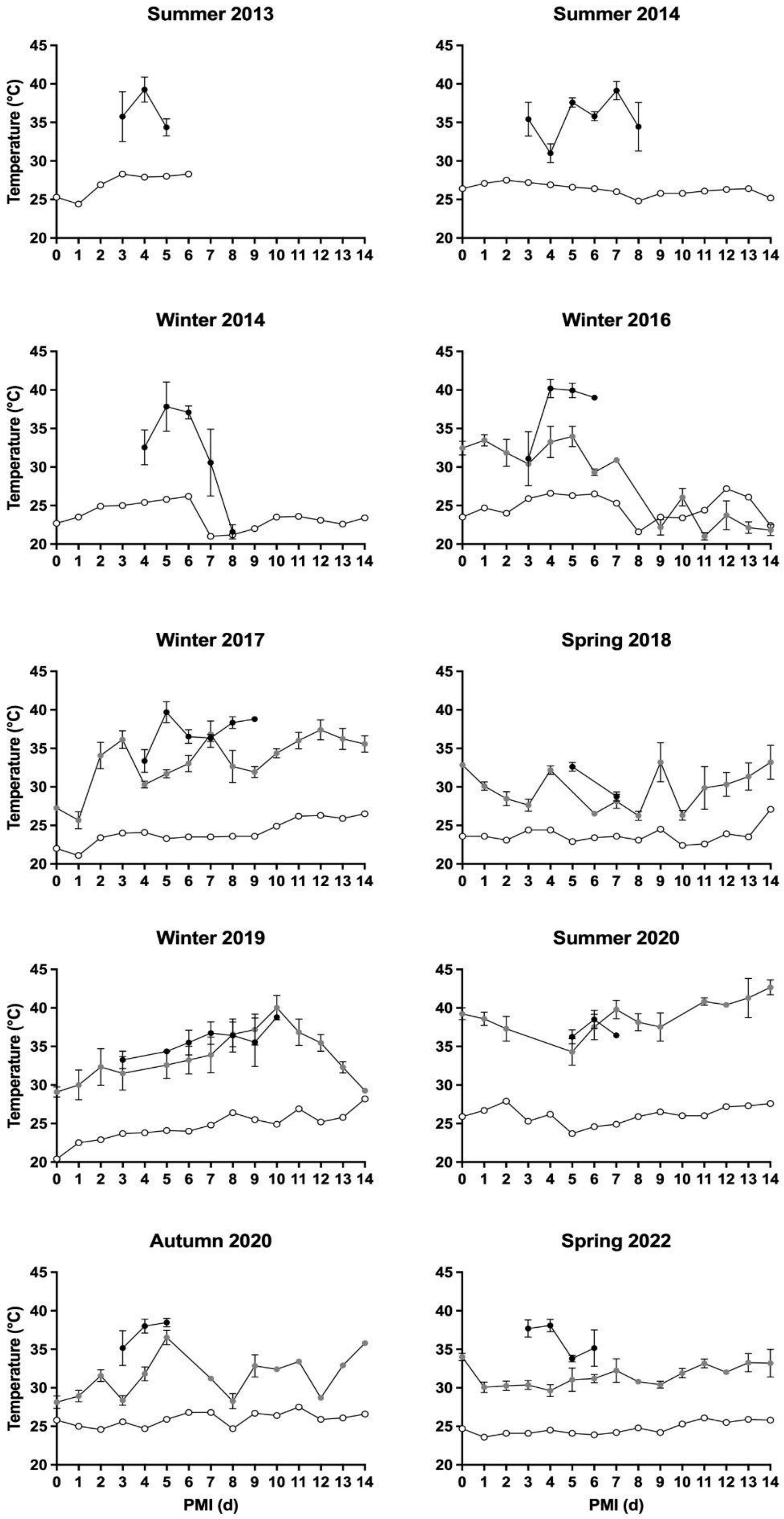

Figure S5. Ambient (○), larval mass (●), and skin (●) temperature associated with pig (*Sus scrofa domesticus*) carcasses at the Chaminade facility in Honolulu, Hawaii, USA. Bars represent standard errors where n = 3.

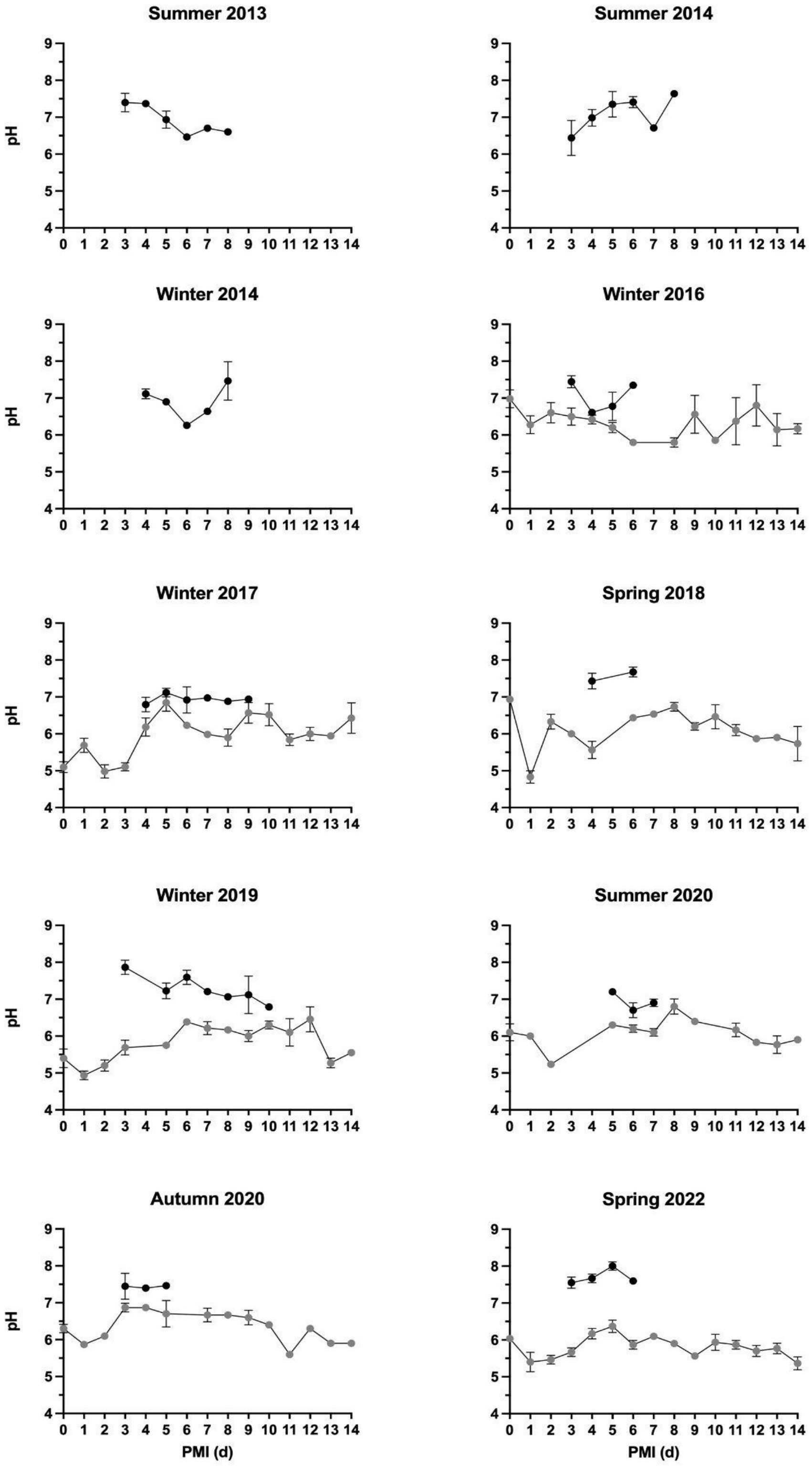

Figure S6. Larval mass (●) and skin (●) pH associated with pig (*Sus scrofa domesticus*) carcasses at theChaminade facility in Honolulu, Hawaii, USA. Bars represent standard errors where n = 3.

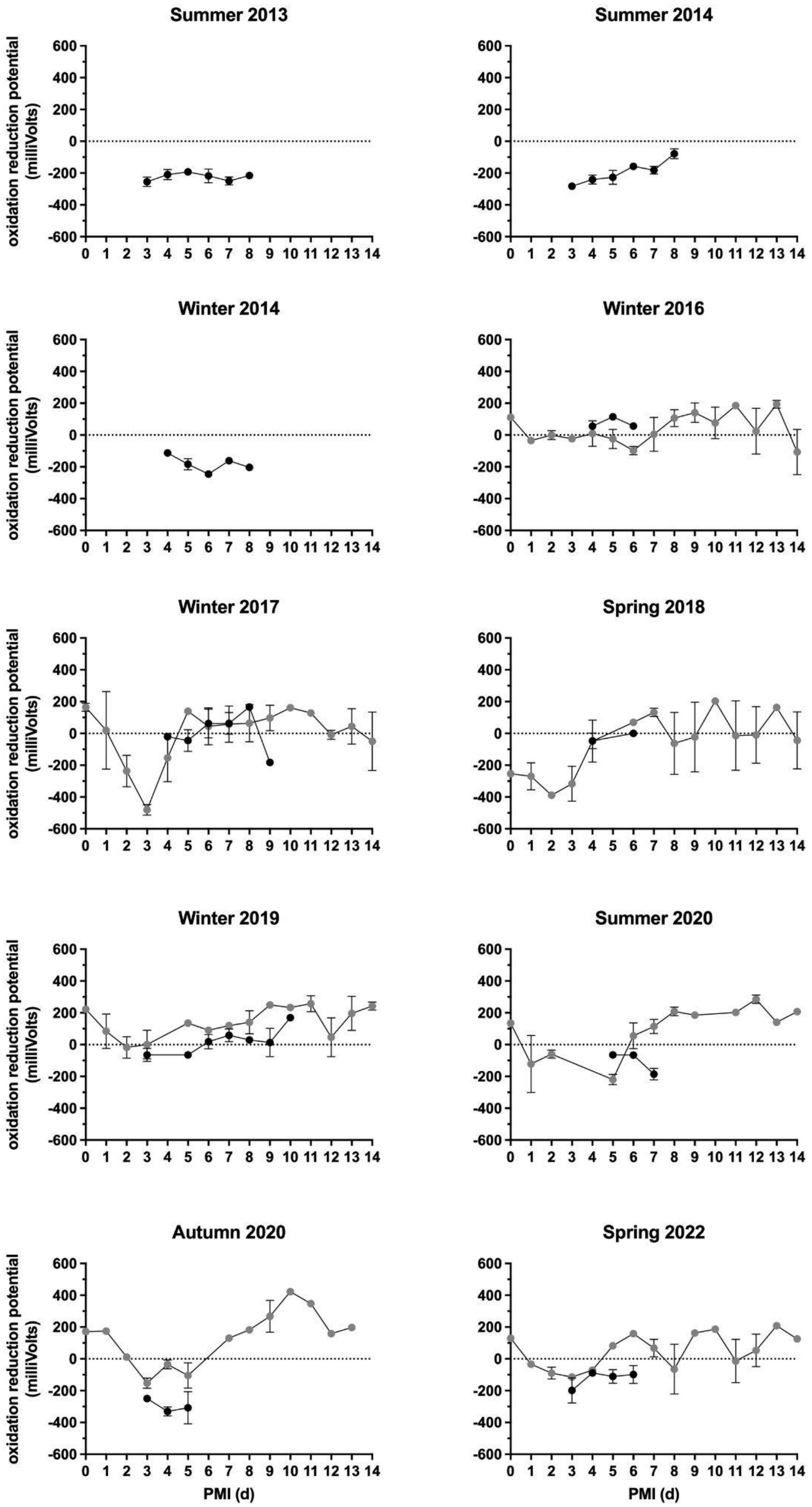

Figure S7. Larval mass (●) and skin (●) oxidation reduction potential (Eh) associated with pig (*Sus scrofa domesticus*) carcasses at the Chaminade facility in Honolulu, Hawaii, USA. Bars represent standard errors where n = 3.

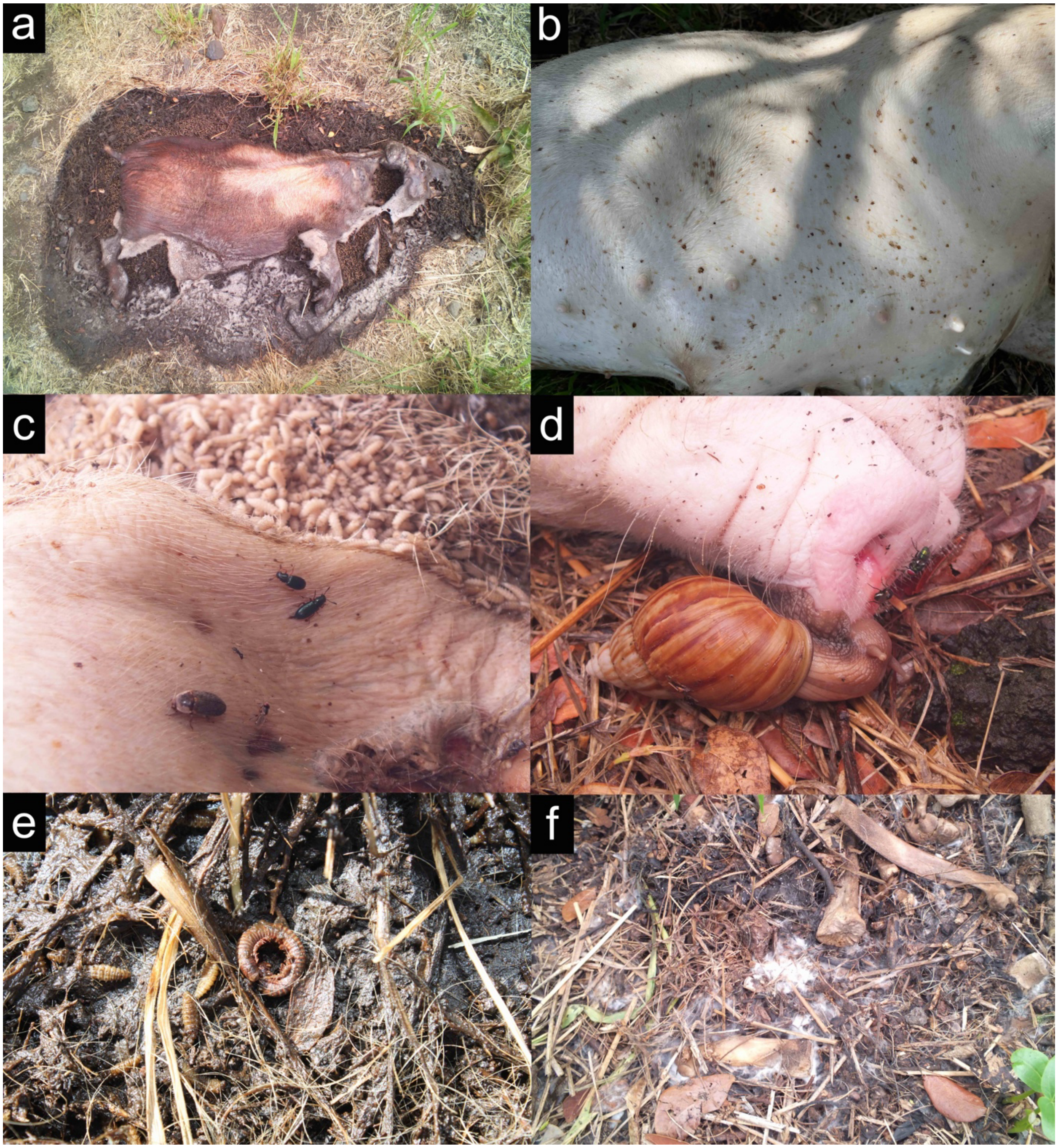

Figure S8. A number of organisms were observed within the Cadaver Decomposition Island (CDI) of pig (*Sus scrofa domesticus*) carcasses at the Chaminade facility in Honolulu, Hawaii, USA. The CDI extended to include areas around each carcass (a), the shape of which was largely determined by slope. All carcasses attracted significant fly activity that included the formation of insect stains (b) similar to those observed with bloodstains. Other insects, including ants, beetles, flies (c) and land snails (d) were observed in the CDI although they were not nearly as conspicuous or abundant as fly larvae. Many of these organisms, particularly fly larvae, do not survive the decomposition process and CDIs consistently included the carcasses of fly larvae and millipedes (e). Hyphae-like structures were also observed in association with some carcasses. These have been observed on the carcass and the soils within the CDI (a, f). Postmortem interval for each CDI: (a) 161 ADD/148 hours, (b) 52 ADD/55 hours, (c) 80 ADD/79 hours, (d) 29 ADD/31 hours, (e) 227 ADD/221 hours, (f) 268 ADD/247 hours.
